# Estimating excess mortality due to female genital mutilation

**DOI:** 10.1038/s41598-023-38276-6

**Published:** 2023-08-16

**Authors:** Arpita Ghosh, Heather Flowe, James Rockey

**Affiliations:** 1 Department of Economics, University of Exeter, Rennes Dr, Exeter, UK; 2 School of Psychology, University of Birmingham, Edgbaston, Birmingham, UK; 3 Department of Economics, University of Birmingham, Edgbaston, Birmingham, UK

**Keywords:** Diseases, Risk factors

## Abstract

Globally, over 200 million women and girls have been subjected to Female Genital Mutilation (FGM). This practice, illegal in most countries, often happens in unsanitary conditions and without clinical supervision with consequent bleeding and infection. However, little is known about its contribution to the global epidemiology of child mortality. We matched data on the proportion of girls of a given age group subject to FGM to age-gender-year specific mortality rates during 1990–2020 in 15 countries where FGM is practised. We used fixed-effects regressions to separate the effect of FGM on mortality-rates from variation in mortality in that country in that year. Using our estimated effect, we calculated total annual excess mortality due to FGM. Our estimates imply that a 50% increase in the number of girls subject to FGM increases their 5-year mortality rate by 0.075 percentage point (95% CI $0\cdot 065$–$0\cdot 085$). This increased mortality rate translates into an estimated 44,320 excess deaths per year across countries where FGM is practised. These estimates imply that FGM is a leading cause of the death of girls and young women in those countries where it is practised accounting for more deaths than any cause other than Enteric Infections, Respiratory Infections, or Malaria.

## Introduction

Female Genital Mutilation, defined by the World Health Organization as involving *the partial or total removal of external female genitalia or other injury to the female genital organs for nonmedical reasons*, is a human rights violation that has affected around 200 million girls and women alive today^1^. FGM is known to lead to obstetric complications^2–4^, reductions in sexual function^5^, and other long-term physical health problems^6^, as well as mental health problems^7,8^. FGM is illegal in most countries where it is practised and partly because of this it typically takes place in a non-sanitary environment and is not performed by clinicians. The available evidence shows that it often leads to severe pain, bleeding, and infection^9^, and sometimes death^2,10,11^. Given that it is performed on so many girls and women the aggregate health impacts of these complications are likely to be large. The WHO estimate the aggregate cost of medical treatment for girls and women after FGM was $1.4 billion in 2018^12^. But, there is no systematic evidence about the role of FGM in the global epidemiology of child mortality. This in part reflects measurement difficulties. Measurement is difficult because FGM is illegal in most countries, because it may not always be identified as the underlying cause, and because the countries in which it is common are low income and have limited state capacity. Our analysis is designed to circumvent these difficulties and provide evidence as to the scale of excess mortality due to FGM. Our main aim is to estimate the effect of FGM on overall mortality rates and to use these estimates to calculate total annual excess mortality due to FGM.

Our approach is simple and is similar in spirit to the approaches used in forensic economics^13,14^. The key idea is that to the extent that FGM leads to death, this should be captured by increases in age-specific mortality rates. To compute these increases, we constructed a dataset combining information on the proportion of girls of a given age subject to FGM in each country with age-gender-year specific mortality rates. We used regression analysis to separate the effects of FGM from other country and year specific sources of mortality. We find that other things being equal, a country in which 50% more girls of a given age were mutilated would be associated with a 0.075 percentage point (95% CI $0\cdot 065 - 0\cdot 085$) increase in their age-group-specific mortality rate, equivalent to an additional 44,320 girls dying a year.

It is important to understand the scale of previous research which has highlighted the many consequences of FGM for girls throughout their lives, something our analysis does not consider. One aspect of this literature is the direct consequences of FGM for women’s sexual pleasure and relationships^15–17^. Communities in which FGM is common may impose costs on women and girls who are uncircumcised. For example recent research^18^, documents the social pressure and punishment, including exclusion from community functions, faced by uncircumcised adult women in North-Eastern Uganda. A key social dimension of FGM is how it impacts on marriage markets. For example, FGM influences women’s marriage opportunities in Western Africa and these practices might persist due to patriarchal culture and institutions^19^. Other research documents a causal relationship between reductions in FGM and increased parental investment in girls’ education in Senegal which is  attributed to the reduced bride price of non-circumcised women^20^. Other work finds, found that reductions in FGM prevalence reduce bride prices in Egypt^21^.

Considered as a whole, this research suggests that the decisions about FGM may often reflect tradeoffs between perceived disadvantages of FGM such as pain, illegality, and expected benefits such reduced social sanctions and a higher bride-price. Our results suggest that higher mortality may also be part of that calculus.

Another stream of the literature explores if and how FGM practices have been decreasing in different regions in Africa, and how attitudes can be changed. In Egypt, there is a large gender gap in the beliefs regarding FGM and boys are more supportive of these practices than girls^22^. On the other hand, other research finds that majority of the variation in these practices are due to household and individual level factors^23^. Women who have experienced FGM first-hand are more likely to support this practice. Similarly, even increased maternal education might not alter the probability of FGM on their girls^24^. Research exploring the reasons behind the persistence of these practices, shows that marriage market pressures might result in high levels of FGM, but even weak regulations banning FGM can reduce it^25^. Other recent research, provides theory and evidence for when there may be an FGM tipping point—that once rates of FGM fall below a certain level the practice rapidly dies out^26^. Information seems to be a key mechanism for reductions in the prevalence of FGM , For example, there have been reductions in FGM rates in Egypt due to increased social media reach^27^. Likewise, showing a movie about different views on FGM can alter attitudes towards FGM^28^.

Our finding that FGM is associated with an increased mortality rate equivalent to around 44,320 excess deaths per year in the countries in our sample, shows how urgent and important the results of this literature are. They suggest that identifying strategies to reduce or eliminate FGM has the potential to save a great number of lives^11,29–34^.

From a broader perspective, the high rate of excess mortality due to FGM in Africa can be seen as an example of the negative consequences of patriarchal institutions. Such institutions inherently have complex origins. In the case of FGM, recent research has drawn links between the historic Red Sea slave trade in the period 1400-1900, the use of infibulation as a ‘guarantee’ of the virginity of girls being sold into harems, and FGM today^35^. Beyond FGM, other recent work has related variation within Africa in attitudes towards, and the prevalence of, gender based violence to both pre-colonial differences in women’s economic role and marriage practices^36^, and differences in colonial experience^37^.

## Data

### Overview

There are no existing data that track the number of deaths due to FGM. Instead, we compute the excess mortality rate associated with FGM. In this section we detail the data and our method to infer excess mortality due to FGM practices. We use the World Population Prospects report^38^ to obtain the mortality data for girls and boys. For the FGM prevalence data in 15 countries, we use the Demographic and Health Surveys^39^. These anonymized data were made available by the DHS following registration. At no point were non-anonymized data held, and all data were handled in accordance with the Declaration of Helsinki. Additionally, we use data from the Orchid Project as an alternative data source, which provides only the typical age at which FGM takes place, but provides data for nine further countries. The following subsections provide more details on the variables we use in this paper. More details are also provided in Appendix A in the supplementary materials.

### Calculating mortality rates

We calculated (separately for girls and boys) the mortality rate at each age in each year. To do this we divide the number of age-gender specific deaths in a given year by the initial population of that age and gender to obtain the mortality rate. For example, if the female population in 2012 aged 4 was 1000, and there were 8 fatalities then this would then imply a mortality rate of 8/1000 = 0.8% for 4-year-old girls in 2012.

### Inferring additional deaths due to FGM

The best available data on FGM is based on the Demographic and Health Surveys (DHS) programme. These surveys collect data not only on whether women have been subjected to FGM but whether their daughters have been, how it was done, etc. Here we use these data to compute for each country the distribution of ages at which FGM occurs. The resulting data as percentages of those mutilated are reported in Table 1.

**Figure 1:**
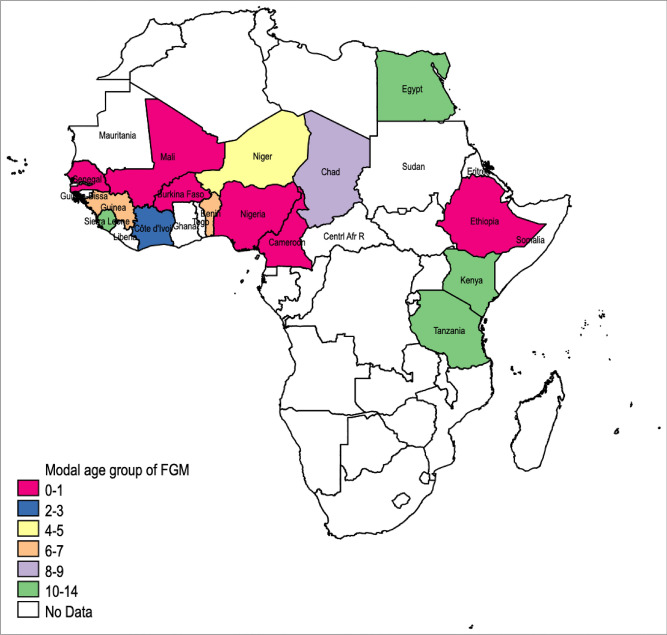
Modal age (for DHS sample) at which FGM occurs in countries in our analysis. *Notes:* This map was created with spmap package^59^ in Stata 16 MP8 by the authors, using shapefiles from openAfrica. This map only shows the 15 countries from DHS samples in colours and modal age groups.

The data show that there is considerable variation across countries in the age at which FGM is performed. In Nigeria, for example, we can see that 93% of the time it is performed whilst the girl is younger than age five, while in neighboring Benin it is more common for it to take place when the girl is up to nine years old, or older. Such differences across neighbouring countries are common; for example Ethiopia and Kenya are quite different in this respect, and there is no obvious pattern. This can be easily seen in Fig. 1 which maps, for Africa only, the countries in which FGM is practised and the modal age at which it takes place. This highlights the variation in the modal age both within and between East Africa and West Africa. In most West African countries FGM occurs by the age of 6–7, with it often happening to girls when they are infants. Moreover, as Table 1 makes clear there is variation in the distribution of ages at which FGM is performed beyond that captured by the mode.

## Methods

To our knowledge there are no existing data that track the number of deaths due to FGM in the period following the procedure. Instead, we exploit variation in the number of girls and young women mutilated and the age at which it takes place to compute the excess mortality rate associated with FGM. To do this we use a linear regression model. Specifically, we run population weighted linear regressions with country and year fixed effects. That is, we run linear regressions including vectors of binary variables for each country and year, or country-year pair. Our linear regression is of the following form:

1 $$\begin{aligned} Mort^{g}_{act}=\alpha + \beta Mort^{b}_{act}+\gamma FGMAge_{{\overline{a}}c}+\lambda _{ct}+\varepsilon _{act} \end{aligned}$$

Where *a* is age, *c* is country, and *t* is calendar year. Our outcome variable, $Mort^g_{act}$ is the mortality rate for girls of age *a*, in country *c*, and year *t*; whereas $Mort^b_{act}$ is the male mortality rate at age *a*, in country *c*, and year *t*. The key variable of interest ${FGM Age}_{{\overline{a}}c}$ is the percentage of girls subject to FGM in age group ${\overline{a}}$ in country *c* as reported in Table 1. The idea is that, since we expect excess mortality associated with FGM to occur at the time of FGM, or in few months after, this variable will capture the additional mortality. We compute heteroskedasticity robust standard errors.

So, in Chad where 1.7% of girls aged 0–4 are subject to FGM, *FGMAge* is equal to 0.017 for that age group, and 0.145 for the 10–14 age-group. We attribute all FGM beyond that age to the age group 15–19. This methodology is appropriate because girls are married before the age of 20 and mutilation precedes marriage in the countries being analysed^40,41^.

Girls’ mortality rates are in general lower than those of boys in the same country of the same age. However, both will vary depending on local conditions such as drought or the disease environment. Thus, including male mortality, $Mort^b_{act}$, will capture such variations which may disproportionately affect children in a given country and year of different ages differently. FGM will also lead to excess deaths during later childbirth^3,4^, but this is difficult to account for and thus our estimates will be conservative in this regard. Likewise, any measurement error in $FGMAge_{ac}$ will attenuate our estimate of $\gamma$, also making our estimates conservative. Our estimate of $\gamma$ is then the increase in mortality amongst girls of the age at which FGM is normal in a given country compared to girls of the same age in other countries where FGM is common but in which it tends to happen at a different age.

Our preferred specification tightens this comparison by also allowing for country-year specific variation in female mortality. The inclusion of $\lambda _{ct}$ allows for variations in the average female-mortality rate in a given country in a given year. This means $\gamma$ will also take into account the mortality rate of women and girls in that country in that year.

Together the inclusion of $Mort^{b}_{act}$ and $\lambda _{ct}$ means that the requirement for an alternative explanation, not captured by our specification, is demanding. Namely, that it (disproportionately) affects girls compared to boys of the ages when FGM takes place, but not girls of other ages.

Given our estimate of increased mortality, ${\hat{\gamma }}$, we can calculate what this implies in terms of total excess mortality by multiplying the excess mortality by the population at risk as follows:

2 $$\begin{aligned} \text{ Deaths } \text{ Due } \text{ to } \text{ FGM } = \text{ Women } \text{ and } \text{ Girls } \text{ at } \text{ Risk } \text{ of } \text{ FGM } \times {\hat{\gamma }} \end{aligned}$$

The number of Women and Girls at Risk of FGM is computed as the number of females in the population in each age group in each country multiplied by the percentage of women and girls subject to FGM at that age in that country aggregated across all the countries we study. That is, the (estimated) number of women and girls directly affected by FGM. This is computed as follows:

3 $$\begin{aligned} \text{ Deaths } \text{ Due } \text{ to } \text{ FGM }&= \sum _{c \in C} \sum _{{\overline{a}} \in {\overline{A}}}\left( FGMAge_{{\overline{a}}c} \times Pop^{g}_{{\overline{a}}c}\right) \times {\hat{\gamma }}. \end{aligned}$$

Where $Pop^{g}_{{\overline{a}}c}$ is the population of women and girls in age group ${\overline{a}}$ in country *c*. Data from the UN Population Division^42^ suggest that there were around 23.5 million women and girls affected in 2020, in the countries we study.

It is important to note that ${\hat{\gamma }}$ is the average increase in mortality associated with FGM but, due to differences in the practice of FGM across countries, the increase may be different in different countries. In general ${\hat{\gamma }}$ will not be the arithmetic average of the effect in individual countries, meaning that Eq. 2 may over- or under-state total excess mortality associated with FGM. To alleviate these concerns, following^43^, in our preferred regression specification we weight by country population size. However, to the extent that the increase of mortality associated with FGM varies within countries, our estimate of ${\hat{\gamma }}$ will be inaccurate, and thus our calculations should be seen as necessarily approximate. Moreover, given that $\hat{\gamma}$, like all estimates, has an associated confidence interval then this implies a confidence interval for the number of excess deaths which we report to convey the degree of statistical uncertainty.

## Results

Table 2 presents estimates of Eq. 1. Moving from left to right the columns report increasingly demanding specifications. To start with, column (1) represents a very simple specification only including the FGM and male mortality variables, and not weighting the data by population size. Column (2) shows the same specification, now weighting by population. Column (3) adds country and year additive fixed effects. That is, we now have binary variables for each country and each year. Column (4) is our preferred specification in which we allow for average female mortality to differ across countries and years in an unrestricted way. That is, in this case we include a binary variable for each country-year pair.

In all specifications excess mortality is positively correlated with $FGMAge_{{\overline{a}}c}$. As expected there is also a large positive correlation $Mort^{b}_{ait}$ reflecting that many causes of death do not discriminate. Likewise, the constant term is negative reflecting that mortality rates are, on average and in the absence of FGM, lower for girls than for boys. Weighting by population size as in column (2) reduces ${\hat{\gamma }}$ by a factor of around two. This perhaps reflects, that in our sample, the average severity of FGM is lower in larger countries or in regions where suitable medical care is more available.

As we introduce country and year, or country-year fixed effects the estimate increases reflecting the more precise comparison. In our preferred specification in column (4) it is around 0.0021 (95% CI $0\cdot 0019 - 0\cdot 024$) implying that, other things being equal, a country in which 50% more girls of a given age were mutilated would be associated with a $0.5\times 0.0021=0.1$ percentage point (95% CI $0\cdot 095 - 0\cdot 12$) increase in their mortality rate. The magnitude of this number is reflected in the associated total number of excess deaths in the countries we study calculated using Eq. 2. Specifically, we have:

4 $$\begin{aligned} 23,520,938 \times 0\cdot 0021 = 44,320 \end{aligned}$$

The implied associated 95% confidence interval is between 38,230 and 50,400 excess deaths a year.

Columns (5)–(8) present a number of robustness checks. Column (5) additionally includes binary variables for each age group allowing for age-specific differences in average female mortality rates. We do not include this in our preferred specification as we expect it in part to be determined both on average, and in a given country, by FGM and thus is a so-called *bad control*^44^. Including these controls, the estimated effect of FGM falls by around two thirds. This is unsurprising as the age controls will be capturing some of the variation due to FGM. But, the implied number of excess deaths due to FGM is still large at over 11,000 per year.

The remaining columns address concern about possible measurement error in $FGMAge_{{\overline{a}}c}$. While the systematic nature of the DHS surveys means that we believe those data are to be preferred, in particular because they provide precise data on the age at which FGM happens and prevalence which is crucial to our approach, we also report results using alternative data from the Orchid Project. These provide coverage of some additional countries but require us to assume that all FGM happens at the same age in a given country. Thus, for example, if 24% of women and girls have suffered FGM then we assume that that happened for all of them, at say, age 14 if that is the most common age.

Column (6) shows that using these alternative data for the same set of countries we obtain similar results; our estimate of $\gamma$ is around 50% smaller than in our preferred specification and there is an associated reduction in implied excess fatalities per year. Column (7) reports results for all the countries covered by the Orchid Project data, additionally including Central African Republic, Eritrea, Ghana, Guinea-Bissau, Liberia, Mauritania, Somalia, Sudan, and Togo. Now the estimate of gamma is larger than in column (6) but still smaller than in column (4). The implied number of excess fatalities is similar, at around 42,000 women and girls per year. This reflects a reduction in the estimated increase in mortality, but also the increased at risk population since here we also include the additional nine countries for which the Orchid Project data are available, which are not covered by the DHS. Table B1 in the Supplementary Materials presents the results of the specifications in columns (2), (3), and (4) using the Orchid Project data for the DHS countries and for all countries covered by the Orchid Project in turn. As a further robustness check, Figure B1, in the Supplementary Materials, presents estimates omitting one country at a time to demonstrate that our results are not driven by any one country. One reason this is important is because the practice of FGM varies by country, with more severe forms of FGM, such as infibulation, being more common in some countries than others^45^.

## Discussion

FGM is a leading cause of death in the countries where it is practiced. Our estimate that 44, 320 girls and young women die each year due to FGM is suggestive that FGM belongs in the first rank of causes of death in Africa. As a comparison, there were around 968,000 combat deaths in Africa over the 20 years from 1995, but that there were more than 3.4 times as many excess infant deaths associated due to armed conflict, suggesting around 165,000 deaths per year^46^.

**Figure 2:**
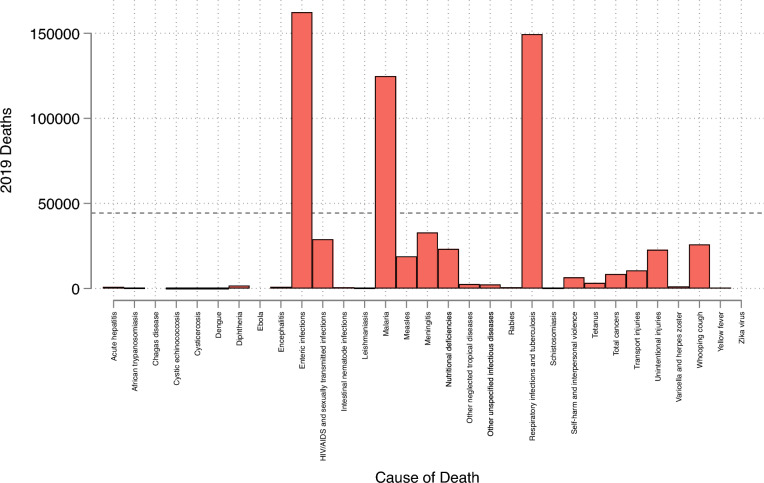
Other Causes of Death. *Notes:* The figure reports the total annual number of deaths, in the countries we study, by different causes. Total annual deaths due to FGM are depicted as the horizontal dashed line. Data are from the Institute for Health Metrics and Evaluation Global Burden of Disease database.

In the countries we study, Malaria and other neglected tropical diseases accounted for the deaths of around 130,000 women girls under the age of 20 in 2019. Notably, however, restricting our attention to those aged 5–19 there were around 12,000. Figure 2 compares total annual deaths, in the countries we study, due to FGM (the dashed horizontal line) with all other causes of death (note, that these totals will include deaths due ultimately to FGM). The leading causes of death in the countries in our sample are Enteric infections, Respiratory infections, and Malaria. Our estimates imply however, that FGM is responsible for more deaths of girls than any other cause including HIV/AIDS, Measles, Meningitis, starvation (nutritional deficiencies), injuries, or whooping cough. On this basis it may be regarded as an urgent social and medical issue alongside its status as a human rights violation.

### Limitations

There are some important caveats associated with this analysis. First, we cannot be sure that the excess deaths are caused by FGM. It could be that there is some other cause that has a very similar pattern across countries and age groups. However, we do not believe this is likely given the robustness of our finding to a range of specifications, data sources, and to leaving out one country at a time. Second, our results are likely an under-estimate. This is because we do not consider consequent obstetric complications and because FGM will be measured with statistical error attenuating the estimated coefficient. Third, there is some evidence that FGM has become less common as NGOs and governments work to discourage it^30,47–52^. While we use the best and most recent data on FGM prevalence, our results describing historic rates of excess mortality associated with FGM may be higher than current rates. On the other hand, rapid population growth and slow cultural change may alternatively mean current rates are in fact higher. Moreover, there is evidence that COVID-19 has led to a resurgence of FGM^47^. Indeed, the UN estimates^53^ an additional 2 million cases of FGM that would otherwise have been prevented if not for Covid-19. In this context, our coefficient estimates suggest these additional cases will be associated with 4000 additional deaths.

An important caveat is that our estimate of the total number of excess deaths is based on the average relationship across the countries studied. While we use population weights to minimise this concern, it does mean that our estimates can only be seen as approximate. In future work it would be valuable to conduct analyses at the sub-national level to alleviate this concern. It would also be very valuable, were such data to become available, to use time-series variation in the rate of FGM, and the age at which it occurs, in each country.

## Conclusions

Our results suggest FGM is a leading cause of death amongst girls and young women in countries where it is practised. This is further evidence of how important the work of those civil-society organisations, governments, and international agencies that seek to eliminate FGM is. However, as the increase in the number of FGM cases during Covid-19 suggests, lasting change will require changing attitudes towards FGM in the communities where it is practised. On one hand there is cause for optimism: work on non-communicable diseases shows that effective interventions are possible^54^. On the other, change in patriarchal attitudes have often lagged other societal change^55^. Experience to date suggests that, whilst progress is being made, it has often been slow^56^. FGM remains legal in five of the 28 countries where it is most commonly practiced^57^. An important first step would be for FGM to be made illegal in those countries, given that legal change can lead to cultural change^58^.

### Directions for future research

Our analysis has focussed on variation at the national level. Yet, the prevalence of FGM often varies a great deal within countries. It would be valuable for future research to study how excess mortality varies within countries between areas in which FGM is differentially prevalent. The key difficulty in undertaking such research would be to assemble suitably precise data on both FGM and mortality rates. Likewise, as work continues by governments and NGOs to reduce the prevalence of FGM it would be valuable to study the impact of the consequent reductions in FGM on mortality. Finally, in this paper we have treated FGM as a single practice. In reality the practice of FGM varies, and in future work, if the data allowed, it would be valuable to understand whether the different forms of FGM are associated with different rates of excess mortality.

**Table 1:** Age at which FGM occurs by Country.

Country	Age groups							
	0–4	5–9	10–14	15–19	20–24	25+	Total	Women/girls **Circumcised (%)**
Benin	32.7	56.6	9	1.6	0.1	0	478	7.3
Burkina Faso	59.8	36	3.6	0.5	0	0	3610	75.8
Cameroon	57.4	36	6.6	0	0	0	18	1.4
Chad	12.1	58.5	27.4	1.8	0.2	0	898	38.4
Cote D’Ivoire	50.9	32.5	14.3	2.3	0	0	357	38.2
Egypt	0.7	5.2	29.9	56.5	0	0	–	92.3
Ethiopia	64.8	21	10.3	3.7	0.3	0	6615	65.2
Guinea	18.7	64.2	15.9	1.1	0	0	5081	94.5
Kenya	2.9	42	38.3	16.4	0.4	0	971	21
Mali	77.8	18.3	3.6	0.2	0	0	9866	88.6
Niger	69.1	22.3	7.3	1.4	0	0	298	2
Nigeria	92.6	3.9	1.4	1.5	0.5	0.1	4138	19.5
Senegal	83.3	14.1	2.3	0.2	0	0	1745	25.2
Sierra Leone	0.3	4.8	16.7	61.2	0	0	–	83
Tanzania	34.5	26.2	26.1	12.5	0.6	0	727	10
**Total**	68.1	20.2	8.4	3.0	0.3	0.0	100 (34802)	–

*Notes:* Proportions are calculated using data from the FGC module of the DHS. In those countries where multiple survey waves are available, data for the most recent wave are reported. Population weights are used such that data are nationally representative in each case. Total frequency in the 8th column is absolute, unweighted, number of respondents. The row totals do not include Egypt and Sierra Leone as the individual level survey data were not available. For these countries data was instead obtained via the DHS Stat Compiler https://www.statcompiler.com/en/ tool.

**Table 2:** The impact of FGM on female mortality rates.

	(1)	(2)	(3)	(4)	(5)	(6)	(7)
${FGM Age}_{{\overline{a}}c}$	0.0036***	0.0015***	0.0021***	0.0021***	0.0006***	0.0007***	0.0011***
	(0.0001)	(0.0001)	(0.0001)	(0.0002)	(0.0002)	(0.0001)	(0.0001)
$\text{ Mort}^{m}_{ait}$	0.8886***	0.8714***	0.8666***	0.8664***	0.8922***	0.8662***	0.8664***
	(0.0009)	(0.0019)	(0.0017)	(0.0017)	(0.0056)	(0.0018)	(0.0016)
$\alpha$	– 0.0007***	– 0.0000*					
	(0.0000)	(0.0000)					
Country and Year Effects	No	No	C + Y	C $\times$ Y	C $\times$ Y	C $\times$ Y	C $\times$ Y
Weighted	No	Yes	Yes	Yes	Yes	Yes	Yes
FGM Measure	Main	Main	Main	Main	Main	Orchid	Orchid+
Age Effects	No	No	No	No	Yes	No	No
Excess FGM Deaths	74.35	31.31	44.31	44.32	12.82	29.11	42.19
Excess FGM CI UL	79.25	36.84	50.33	50.40	19.34	18.57	24.71
Excess FGM CI LL	69.45	25.79	38.29	38.23	6.29	12.13	19.79
Adjusted R square	0.99	0.99	0.99	0.99	0.99	0.99	0.99
Observations	42315	42315	42315	42315	42315	42315	67704

*Notes:* The dependent variable is the female death rate amongst those in age *a* in country *i* in year *t*. $${FGM Age}_{{\overline{a}}c}$$ is the percentage of girls subject to FGM in age group *a* in country *i*. $$\text{ Mort}^{b}_{ait}$$ is the male mortality rate in age-group *a* in country *i* in year *t*. $$C+Y$$ denotes that the regression model additionally includes country and year specific binary variables. $$C \times Y$$ denotes that the model includes a binary variable for each country year combination. The *main* FGM measure is the percentage of girls subject to FGM in age group *a* in country *i* subject to FGM based on the DHS surveys (15 countries). *Orchid* represents that we use data from the Orchid Project instead of the DHS. *Orchid+* denotes that we additionally include those countries for which there are Orchid Project data but not DHS data on FGM (9 additional countries). Age Controls indicates that binary variables for each age are additionally included. Weighted denotes that countries are weighted by populations such that the results are representative of the population as a whole. Robust standard errors in parentheses. Excess FGM CI UL (LL) is the upper (lower) limit of the 95% confidence interval of the number of excess deaths due to FGM. $$*p < 0.1$$; $$**p < 0.05$$; $$***p < 0.01$$. Data are from 1990 to 2020 for mortality.

### Supplementary Information

Supplementary Information.

## Data Availability

All data generated or analysed during this study are included in this published article [and its supplementary information files].
