# Supplementary material for: Estimating excess mortality due to female genital mutilation
Source: Sci Rep. 2023 Aug 16;13:13328. doi: 10.1038/s41598-023-38276-6 (PMC10432559; doi:10.1038/s41598-023-38276-6)
Supplement: Supplementary file 1 — Supplementary Information. [file 41598_2023_38276_MOESM1_ESM.pdf]

Estimating Excess Mortality due to Female Genital Mutilation – Supplementary Materials

A Data

A.1 FGM prevalence data:

We obtained FGM prevalence data from the Demographic and Health Survey (DHS) FGC supplementary module. We concentrate this study on 13 countries for which DHS data are available (the module was not used in countries where FGM is not prevalent). Amongst these countries, Cameroon and Chad had a DHS wave only in 2004. The other 11 countries in our study have had DHS waves with FGC module data for two or longer years.

The FGC module in DHS waves is a set of optional questions. However, this is the most comprehensive data source present in this topic. The survey questions cover if the respondents have any daughter who is circumcised, the ways in which FGM has occurred, the age at which FGM occurs, as well as health complications that might have arisen because of the procedure. In our DHS sample we have 34,802 observations across these countries. As these surveys are collected at the family level, we work at that granularity rather than daughter level, which also avoids any excess mortality due to FGM affecting the sample.

We define the FGM variable as follows in this study. In general $AgeFGM_{\overline{a}c}$ is the percentage of girls subject to this practice in age group $\overline{a}$ in country c. This variable captures the prevalence of FGM practices in different age groups. The available sample for Cameroon is very small, thus we use data from the Orchid Project and assume an overall prevalence of 1%,(Orchid Project). For all other countries we take the percentage of girls subjected to FGM at a particular age (in a country) as the measurement.

To avoid concerns about measurement error we also report results using alternative data based on Orchid Project estimates instead of DHS data for the same set of countries. We note that in some cases, notably Egypt, there is a discrepency between the two data sources and this exercise shows that our results are not sensitive to which is preferred. The Orchid Project data cover some additional countries, and we report results both for the original sample and including these countries.

Table A.1: DHS Waves considered for FGM numbers by Country

**Country**	**DHS Wave considered**
Benin	2011-12
Burkina Faso	2010
Cameroon	2004
Chad	2014-15
Cote d'Ivoire	2011-12
Egypt	2014
Ethiopia	2016
Guinea	2018
Kenya	2014
Mali	2018
Niger	Pooled: 1998, 2006, 2012
Nigeria	2018
Senegal	2019
Sierra Leone	2019
Tanzania	2015-16

Notes: Data are from the FGM module of the DHS. Collected from The DHS Program STAT compiler (`http://www.statcompiler.com`). Funded by USAID. November 26 2021

A.2 Mortality data:

Mortality data is obtained from the World Population Prospects by the United Nations Populations Division. We collect both male and female deaths (and population) data (in thousands) by year and single age from these reports during 1990-2020. We then calculate the percentage of female and male mortality by country and year in question for the regressions reported in this paper. The following graph shows the density of death data by gender used in this study. The male mortality at the ages 0-1 is higher than for their female counterparts. In our outcome sample we have 42,315 observations across the 15 countries. We do not include Malawi, Zimbabwe, Zambia, Uganda, and South Sudan as these countries have no known statistics on FGM. We also exclude South Africa from our analysis as FGM practice data is unknown and it

Table A.2: Orchid Project data considered as alternative prevalence measure in this study

Country	Year (if known)	Source Source	Prevalance (%)	By which age (group) most FGM
**Benin**		UNICEF, DHS and MICS	0.09	14
**Burkina Faso**	2010	UNFPA-UNICEF	0.68	14
**Cameroon**	2004	28 Too Many	0.01	5-9
**Central African Republic***		28 Too Many	0.24	10-14
**Chad**		28 Too Many	0.38	5-9
**Côte d'Ivoire**		28 Too Many	0.37	0-4
**Egypt**	2015	UNFPA-UNICEF	0.87	8-13
**Ethiopia**		UNFPA-UNICEF	0.65	5-9
**Eritrea***		UNFPA-UNICEF	0.83	0-4
**Ghana***	2011, 2014	28 Too Many, MICS, PRB	0.04	0-4
**Guinea**		UNFPA-UNICEF	0.97	5-9
**Guinea-Bissau***		UNFPA-UNICEF	0.45	10-14
**Kenya**	2014	UNFPA-UNICEF, DHS	0.21	11-19
**Liberia***	2013	28 Too Many, DHS	0.5	15-19
**Mali**	2010	UNFPA-UNICEF, MICS	0.83	0-4
**Mauritania***		UNFPA-UNICEF	0.67	0-4
**Niger**	2006	28 Too Many	0.02	0-4
**Nigeria**	2013	UNFPA-UNICEF, DHS	0.19	0-4
**Senegal**	2015-2016	UNFPA-UNICEF, DHS	0.23	0-4
**Sierra Leone**	2013	28 Too Many, DHS	0.9	10-14
**Somalia***		UNFPA-UNICEF	0.98	5-9
**Sudan***		UNFPA-UNICEF, 28 Too Many	0.87	10-14
**Tanzania**		28 Too Many	0.1	0-4, 5, past 13
**Togo***		28 Too Many	0.05	0-4, 10-14

Notes: Data are from the Orchid Project (where does FGC happen). Collected: November 30 2021. The asterisk denote countries for which Orchid data are available but DHS data are not. We exclude data included in the Orchid Project for which no prevalence data are available, including Zambia and Malawi.

is much richer than other countries in our sample.

Figure A.1: World Population Prospects - Mortality distribution by gender

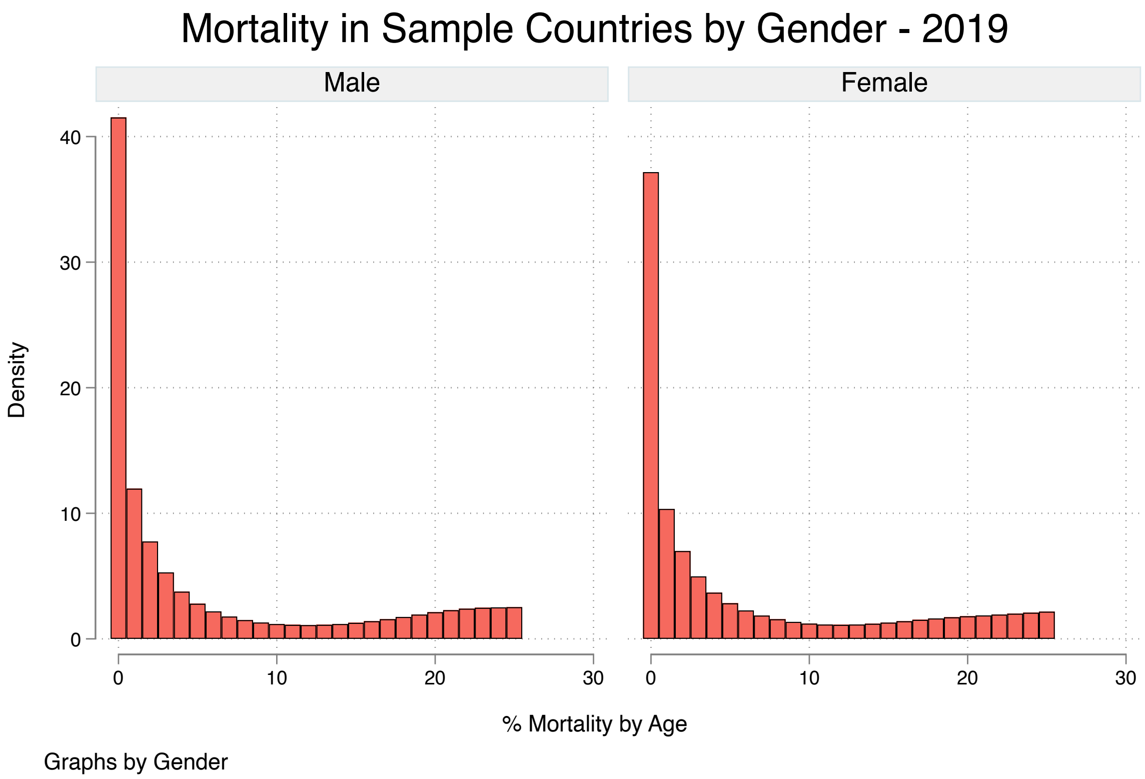

B Additional Results

Table B.1: The impact of FGM on female mortality (Alternative Measures)

	(1)	(2)	(3)	(4)	(5)	(6)
Age FGM$_{ai}$	0.0003***	0.0008***	0.0007***	0.0003***	0.0011***	0.0011***
	(0.0001)	(0.0001)	(0.0001)	(0.0001)	(0.0001)	(0.0001)
Mort$^{m}_{ait}$	0.8713***	0.8663***	0.8662***	0.8712***	0.8664***	0.8664***
	(0.0019)	(0.0017)	(0.0018)	(0.0017)	(0.0015)	(0.0016)
α	0.0000			−0.0001***		
	(0.0000)			(0.0000)		
Country and Year Effects	No	C+Y	C × Y	No	C+Y	C × Y
Weighted	Yes	Yes	Yes	Yes	Yes	Yes
FGM Measure	Orchid	Orchid	Orchid	Orchid+	Orchid+	Orchid+
Age Effects						
Excess FGM Deaths	13.51	30.72	29.11	11.51	42.95	42.19
Excess FGM CI UL	10.65	19.46	18.57	8.56	25.14	24.71
Excess FGM CI LL	3.60	12.94	12.13	3.58	20.16	19.79
Adjusted R square	0.99	0.99	0.99	0.99	0.99	0.99
Observations	42315	42315	42315	67704	67704	67704

The dependent variable is the female death rate amongst those of age a in country c in year t. FGM Age$_{\overline{a}c}$ is the percentage of girls subject to FGM in age group $\overline{a}$ in country c. Mort$^{m}_{cit}$ is the male mortality rate in at age a in country c in year t. $C + Y$ denotes that the regression model additionally includes country and year specific binary variables. $C \times Y$ denotes that the model includes a binary variable for each country year combination. The *main* FGM measure is the percentage of girls subject to FGM in age group $\overline{a}$ in country c subject to FGM based on the DHS surveys. *Orchid* represents that we use data from the Orchid Project instead of the DHS. *Orchid+* denotes that we additionally include those countries for which there are Orchid Project data but not DHS data on FGM. Age Controls indicates that binary variables for each age are additionally included. Weighted denotes that countries are weighted by populations such that the results are representative of the population as a whole. Heteroskedasticity robust standard errors in parentheses. Excess FGM CI UL (LL) is the upper (lower) limit of the 95% confidence interval of the number of excess deaths due to FGM. Mortality data are for the period 1990-2020. $*p < 0.1$, $**p < 0.05$, $***p < 0.01$.

Table B.2: The impact of FGM on female mortality - Alternative DHS Waves for 4 countries

	(1)	(2)	(3)	(4)	(5)
Age FGM_{ai}	0.0038***	0.0017***	0.0023***	0.0023***	0.0008***
	(0.0001)	(0.0001)	(0.0001)	(0.0001)	(0.0002)
Mort^{m}_{ait}	0.8886***	0.8715***	0.8668***	0.8666***	0.8919***
	(0.0009)	(0.0019)	(0.0017)	(0.0018)	(0.0056)
α	−0.0007***	−0.0001**			
	(0.0000)	(0.0000)			
Country and Year Effects	No	No	C + Y	C × Y	C × Y
Weighted	No	Yes	Yes	Yes	Yes
FGM Measure	Main	Main	Main	Main	Main
Age Effects	No	No	No	No	Yes
Excess FGM Deaths	79.45	35.99	48.20	48.19	16.47
Excess FGM CI UL	84.58	41.48	53.85	53.85	23.35
Excess FGM CI LL	74.33	30.51	42.55	42.53	9.60
Adjusted R square	0.99	0.99	0.99	0.99	0.99
Observations	42315	42315	42315	42315	42315

The dependent variable is the female death rate amongst those of age a in country c in year t. FGM $\text{Age}_{\overline{a}c}$ is the percentage of girls subject to FGM in age group $\overline{a}$ in country c. Mort^{m}_{cit} is the male mortality rate in at age a in country c in year t. The *main* FGM measure is the percentage of girls subject to FGM in age group $\overline{a}$ in country c subject to FGM based on the DHS surveys. Mortality data are for the period 1990-2020. We use the rate of age-group-specific rate of FGM for these four countries using instead the earliest available DHS data and reproduce the main specifications in Table 2. Specifically the changes are we take 2012 DHS wave numbers for Guinea and Mali 2012, 2013 for Nigeria, and 2014 for Senegal. For Senegal even though earlier DHS surveys are available, 2014 is the first wave with comprehensive data on the age groups at which girls are subjected to FGM. $*p < 0.1$, $**p < 0.05$, $***p < 0.01$.

Table B.3: The impact of FGM on female mortality - without Cameroon and Niger

	(1)	(2)	(3)	(4)	(5)	(6)	(7)
Age FGM_{ai}	0.0032***	0.0010***	0.0019***	0.0019***	0.0001	0.0008***	0.0011***
	(0.0001)	(0.0001)	(0.0002)	(0.0002)	(0.0002)	(0.0001)	(0.0001)
$Mort^{m}_{ait}$	0.8794***	0.8677***	0.8628***	0.8626***	0.8897***	0.8620***	0.8628***
	(0.0009)	(0.0021)	(0.0018)	(0.0019)	(0.0062)	(0.0020)	(0.0017)
α	-0.0006***	0.0000					
	(0.0000)	(0.0000)					
Country and Year Effects	No	No	C + Y	C × Y	C × Y	C × Y	C × Y
Weighted	No	Yes	Yes	Yes	Yes	Yes	Yes
FGM Measure	Main	Main	Main	Main	Main	Orchid	Orchid+
Age Effects	No	No	No	No	Yes	No	No
Excess FGM Deaths	67.67	20.54	39.45	39.35	2.38	30.63	43.01
Excess FGM CI UL	72.32	26.53	46.34	46.43	9.77	19.71	25.65
Excess FGM CI LL	63.01	14.55	32.57	32.27	-5.01	13.31	20.72
Adjusted R square	0.99	0.99	0.99	0.99	0.99	0.99	0.99
Observations	36673	36673	36673	36673	36673	36673	62062

The dependent variable is the female death rate amongst those of age a in country c in year t. FGM $Age_{\overline{a}c}$ is the percentage of girls subject to FGM in age group $\overline{a}$ in country c. $Mort^{m}_{cit}$ is the male mortality rate in at age a in country c in year t. The *main* FGM measure is the percentage of girls subject to FGM in age group $\overline{a}$ in country c subject to FGM based on the DHS surveys. Mortality data are for the period 1990-2020. We drop Cameroon and Niger from this sample and rerun the main analyses reported in Table 2. $*p < 0.1$, $**p < 0.05$, $***p < 0.01$.

Figure B.1: Country Specific Regressions

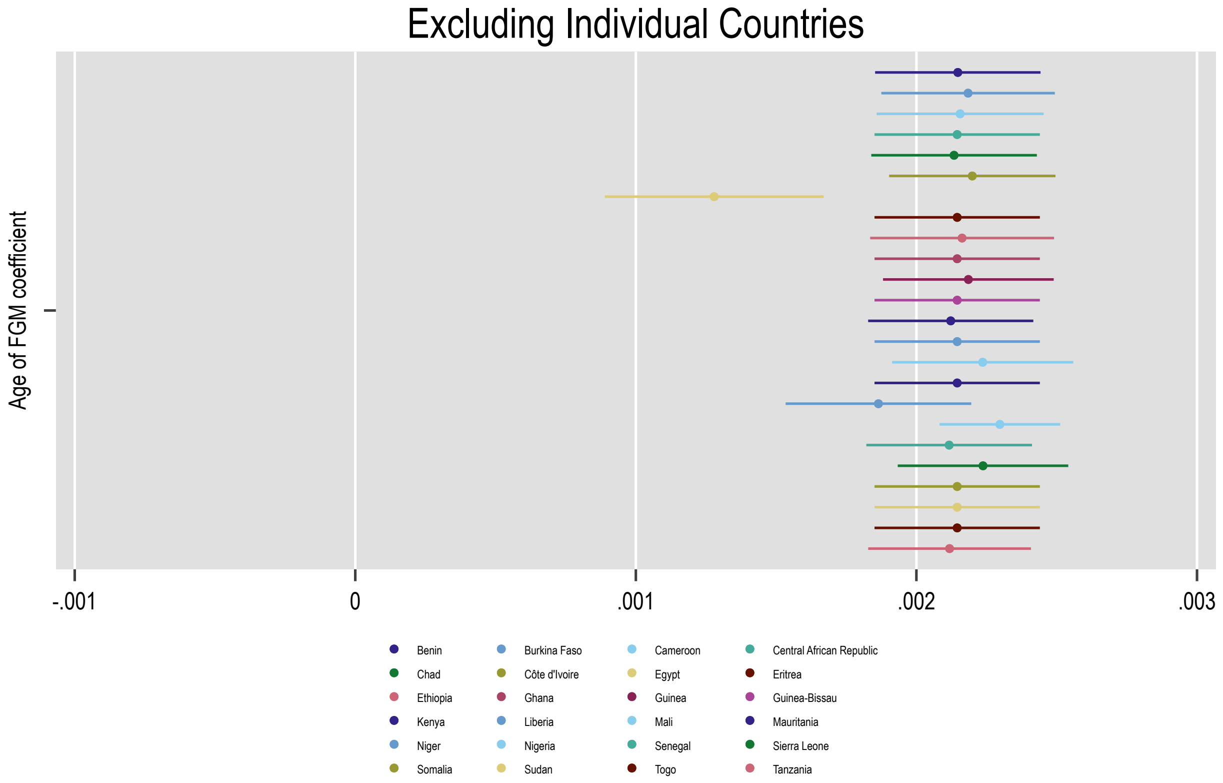

Notes: The figure reports the estimated coefficient for our preferred specification (column 4, in Table 2) excluding one of the countries at a time. Thus, the reported estimate of γ excluding Benin, for example, is the dark blue point at the top of the plot. The associated 95% confidence intervals are depicted by the horizontal lines.

C FGM countries

Table C.1: Modal Age at which FGM occurs by Country

Country	Modal age of FGM	Source
Benin	10	DHS - FGC module
Burkina Faso	12	DHS - FGC module
Cameroon	12	DHS - FGC module
Chad	13	DHS - FGC module
Cote d'Ivoire	10	DHS - FGC module
Egypt	12	Orchid Project
Ethiopia	20	DHS - FGC module
Guinea	12	DHS - FGC module
Kenya	18	DHS - FGC module
Mali	15	DHS - FGC module
Niger	11	DHS - FGC module
Nigeria	18	DHS - FGC module
Senegal	10	DHS - FGC module
Sierra Leone	10-14	Orchid Project
Tanzania	17	DHS - FGC module

Notes: Where data are not available from DHS-FGC survey data are taken from the Orchid Project.

Table C.2: Age at which FGM occurs by Country (Unweighted)

	Age at FGM						
**Country**	**0-4**	**5-9**	**10-14**	**15-19**	**20-24**	**25+**	**Total**
Benin	31.0	57.3	10.3	1.3	0.2	0.0	100.0
Burkina Faso	61.2	34.5	3.7	0.6	0.0	0.0	100.0
Cameroon	55.6	38.9	5.6	0.0	0.0	0.0	100.0
Chad	11.2	57.2	30.0	1.3	0.2	0.0	100.0
Côte d'Ivoire	52.4	32.5	12.6	2.5	0.0	0.0	100.0
Ethiopia	67.1	20.0	10.3	2.4	0.2	0.0	100.0
Guinea	19.4	64.4	15.1	1.0	0.0	0.0	100.0
Kenya	7.7	47.6	30.8	13.6	0.3	0.0	100.0
Mali	78.8	17.5	3.4	0.2	0.0	0.0	100.0
Niger	69.5	24.2	5.4	1.0	0.0	0.0	100.0
Nigeria	93.0	3.6	1.5	1.5	0.3	0.0	100.0
Senegal	84.4	13.6	1.8	0.1	0.0	0.1	100.0
Tanzania	36.5	25.2	25.7	12.1	0.6	0.0	100.0
**Total**	62.4	27.5	8.3	1.6	0.1	0.0	100.0

Notes: Data are solely from the FGC module of the DHS.
